# Complete mitochondrial genome of *Cladosporium* zixishanense sp. nov. YFCC 8620 isolated from the spider in Yunnan, southwestern China

**DOI:** 10.1080/23802359.2019.1699462

**Published:** 2019-12-13

**Authors:** Yao Wang, Yanfang Liu, Guodong Zhang, Mingxi Zhang, Kongfu Zhu, Yuanbing Wang, Hong Yu

**Affiliations:** a Yunnan Herbal Laboratory, School of Life Sciences, Yunnan University, Kunming, China;; b The International Joint Research Center for Sustainable Utilization of Cordyceps Bioresources in China and Southeast Asia, Yunnan University, Kunming, China;; c The Research Center of Cordyceps Development and Utilization of Kunming, Yunnan Herbal Biotech Co. Ltd, Kunming, China

**Keywords:** Cladosporium, mitochondrial genome, phylogenetic analysis

## Abstract

The genus *Cladosporium* is one of the largest and most heterogeneous genera of hyphomycetes. However, little is known about its mitogenome. Here, we first report the complete mitogenome of *Cladosporium* based on the Illumina sequencing data of *Cladosporium zixishanense* sp. nov., which was isolated from the spider. The mitogenome of *C. zixishanense* is composed of a circular DNA molecule with the total length of 37,197 bp, which includes 14 protein-coding genes (PCGs), 2 ribosomal RNA (rns and rnl) genes, 2 ORFs (ORF199 and ORF138), and 26 transfer RNA (tRNA) genes. The overall base composition is 34.7% A, 34.2% T(U), 15.6% C, 15.5% G, with a GC content of 31.1%. Phylogenetic analysis revealed that *C. zixishanense* is located in the order Capnodiales (Dothideomycetes) and forms a separate clade with strong statistical support.

The genus *Cladosporium* belongs to the family Cladosporiaceae (Dothideomycetes), which comprises 845 records in the Index Fungorum (http://www.indexfungorum.org/Names/Names.asp). Species of *Cladosporium* distribute worldwide and are commonly encountered on all kinds of plant, fungal and other debris, are frequently isolated from soil, food, paint, textiles and other organic matters or colonize as secondary invaders leaf lesions caused by plant pathogenic fungi (Bensch et al. 2012). The majority of species in *Cladosporium* have been intensively investigated by using the polyphasic approaches to determine their species identity. However, little is known about the mitogenome of the genus *Cladosporium*, which is one of the largest and most heterogeneous genera of hyphomycetes. During investigating the entomopathogenic fungi in Yunnan province of China, a fungal strain YFCC 8620 was isolated from the dead spider. Based on the morphological and nuclear gene phylogenetic evidence, the strain YFCC 8620 was identified as a new species of *Cladosporium*, namely *Cladosporium zixishanense* sp. nov., and its description will be published elsewhere. This study is the first to report the complete mitogenome of *Cladosporium* and its phylogenetic analysis with other related fungi.

*Cladosporium zixishanense* YFCC 8620 was isolated from the dead spider collected from the Zixishan Forest Park, Chuxiong city, Yunnan province in southwestern China (25°0′34.20″N, 101°24′42.54″E, alt. 2310 m). The strain was deposited at the Yunnan Fungal Culture Collection (YFCC), Yunnan University. Culture on PDA medium at 25 °C for 15 days were prepared to extract total genomic DNA using DNeasy Plant Genomic DNA Purification Mini Kit (QIAGEN). The whole-genome sequencing was conducted by Novogene Co., Ltd. (Beijing, China) on the Illumina sequencing platform (HiSeq-PE150). The 350 bp paired-end libraries were prepared with a sequencing depth of 100 × and data volume of 3 G. We employed the software SPAdes v. 3.11.0 in assembling the mitogenomic sequences of highquality data (Bankevich et al. 2012). The mitochondrial genome was annotated using MFannot tool and ARWEN web server, combined with artificial correction technology. To drew the mitogenomic circular map, the Organellar Genome DRAW tool was used (Lohse et al. 2007).

The annotated mitogenome of *C. zixishanense* was submitted to GenBank under accession No. MN 657180. The total length of this circular mitogenome is 37,197 bp, containing 14 protein-coding genes (PCGs), 2 ribosomal RNA (rns and rnl) genes, 2 ORFs (ORF199 and ORF138), and 26 transfer RNA (tRNA) genes. The total length of the 14 PCGs (atp6, 8–9, cob, cox1–3, nad1–6, and nad4L) is 12,983 bp. The lengths of 26 transfer RNA (tRNA) genes are ranging from 71 to 88 bp, and the sizes of rns and rnl are 1643 bp and 3668 bp, respectively. The sizes of ORF199 and ORF138 are 600 bp and 957 bp, respectively. The overall base composition is as follows: 34.7% A, 34.2% T(U), 15.6% C, 15.5% G, with a GC content of 31.1%.

The 14 PCGs data were obtained from 22 mitogenomes downloaded from NCBI and were aligned using MUSCLE (Edgar 2004). Phylogenetic analysis based on the Bayesian inference (BI) method with the software MrBayes v.3.1.2 was performed to determine the phylogenetic position of *C. zixishanense* (Ronquist and Huelsenbeck 2003). The phylogenetic tree revealed that *C. zixishanense* was located in the order Capnodiales (Dothideomycetes) and formed a separate clade with high credible support by BI posterior probabilities (BI-PP = 100%) (Figure 1).

**Figure 1.**
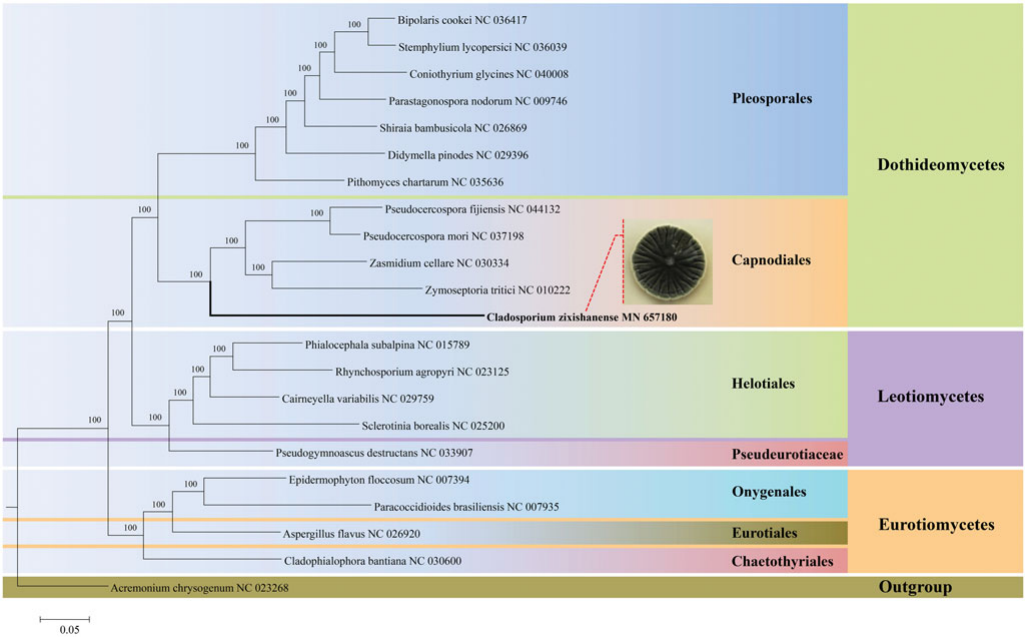
Phylogenetic relationships among 22 species of Ascomycota inferred from the concatenated mitochondrial protein-coding genes (PCGs). The 14 PCGs include subunits of the respiratory chain complexes (*cob*, *cox1*, *cox2*, *cox3*), ATPase subunits *(atp6, atp8, atp9*), NADH: quinone reductase subunits (*nad1*, *nad2*, *nad3*, *nad4*, *nad4L*, *nad5*, *nad6*). The phylogenetic tree is constructed by Bayesian inference (BI) and posterior probabilities are shown above internodes.

## References

[CIT0001] Bankevich A, Nurk S, Antipov D, Gurevich AA, Dvorkin M, Kulikov AS, Lesin VM, Nikolenko SL, Pham S, Prjibelski AD, et al. 2012. SPAdes: a new genome assembly algorithm and its applications to single-cell sequencing. J Comput Biol. 19(5):455–477. 22506599 10.1089/cmb.2012.0021 PMC3342519

[CIT0002] Bensch K, Braun U, Groenewald JZ, Crous PW. 2012. The genus *Cladosporium*. Stud Mycol. 72:1–401. 22815589 10.3114/sim0003 PMC3390897

[CIT0003] Edgar RC. 2004. MUSCLE: multiple sequence alignment with high accuracy and high throughput. Nucleic Acids Res. 32(5):1792–1797. 15034147 10.1093/nar/gkh340 PMC390337

[CIT0004] Lohse M, Drechsel O, Bock R. 2007. Organellargenomedraw (Ogdraw): a tool for the easy generation of high-quality custom graphical maps of plastid and mitochondrial genomes. Curr Genet. 52(5–6):267–274. 17957369 10.1007/s00294-007-0161-y

[CIT0005] Ronquist F, Huelsenbeck JP. 2003. MrBayes 3: bayesian phylogenetic inference under mixed models. Bioinformatics. 19(12):1572–1574. 12912839 10.1093/bioinformatics/btg180

